# High‐Density Lipoprotein‐Specific Phospholipid Efflux (HDL‐SPE) Assay in Mice

**DOI:** 10.1002/mnfr.70492

**Published:** 2026-05-08

**Authors:** Masaki Sato, Edward B. Neufeld, Masato Hamasaki, Alan T. Remaley, Kazuhiko Kotani

**Affiliations:** ^1^ Division of Community and Family Medicine and Department of Clinical Laboratory Medicine Jichi Medical University Shimotsuke‐City Tochigi Japan; ^2^ Future Technology Research Department Eiken Chemical Co., Ltd. Shimotsuga‐gun Tochigi Japan; ^3^ Lipoprotein Metabolism Laboratory, National Heart, Lung, and Blood Institute National Institutes of Health Bethesda Maryland USA

## Abstract

The recent study by Martínez‐Beamonte et al. provides new insights into how extra virgin olive oil enriched in bioactive compounds may exert anti‐atherogenic effects, by improving high‐density lipoprotein (HDL) function, as determined by the high‐density lipoprotein‐specific phospholipid efflux (HDL‐SPE) assay in Apoe‐deficient mice. We would like to highlight two points. First, the HDL‐SPE assay was validated in human samples, but there is only limited data on how it performs in mice. Second, the role of ApoE in ApoA‐I‐containing HDL may differ substantially between mice and humans. The finding by Martínez‐Beamonte et al. is quite intriguing, but it will be important to extend their findings in mice to human subjects.

We read with great interest the recent article by Martínez‐Beamonte et al. [1]. This well‐done study provides new insights into how extra virgin olive oil enriched in bioactive compounds (EVOO HBC) may exert anti‐atherogenic effects, by improving high‐density lipoprotein (HDL) function, as determined by the high‐density lipoprotein‐specific phospholipid efflux (HDL‐SPE) assay [2]. We would like to raise just two points.

First, the HDL‐SPE assay was validated in human samples by showing a correlation with cardiovascular outcomes, but there is only limited data on how it performs in mice. In our initial work in developing the HDL‐SPE assay, we found by electrophoresis that the transfer of fluorescently labeled phosphatidylethanolamine (PE) from donor particles to mouse plasma lipoproteins is HDL‐specific and ApoA‐I‐dependent [3]. Based on this observation, we then went on to develop a rapid and simple procedure that replaces the electrophoretic separation of HDL by directly measuring the fluorescence of the supernatant after the incubation of human plasma with fluorescent PE‐labeled donor particles [2]. The amount of the fluorescent PE probe in the supernatant of human samples was dependent on the amount of ApoA‐I‐containing HDL, and it yielded results consistent with those obtained by electrophoretic analysis [2]. Our recommendation, however, is that future applications of the HDL‐SPE assay in mouse or other types of nonhuman samples should still try to confirm any result by electrophoretic analysis, until it can be established that direct measurement of fluorescent lipid probe in the supernatant is sufficient.

The other point is that in the current study by Martínez‐Beamonte et al. [1], they fed Apoe‐deficient mice with extra virgin olive oil enriched in bioactive compounds, which showed a paradoxical finding that HDL function was improved despite a decrease in ApoA‐I levels. We previously reported with the HDL‐SPE assay a positive correlation between ApoA‐I levels and HDL functionality [2]. We suspect that this discrepancy may possibly arise from fundamental differences in the structural and functional relationships between ApoA‐I and ApoE in human versus mouse HDL. In humans, ApoA‐I and ApoE are thought to be primarily distributed in distinct HDL subclasses. By two‐dimensional electrophoresis analysis, ApoA‐I and ApoE have distinct migration positions, indicating that they are associated with different HDL subpopulations [4]. Moreover, HDL proteomic studies have revealed that only approximately 9% of ApoE is associated with ApoA‐I‐containing HDL particles [5]. In contrast, in mice, approximately a third of ApoE is associated with ApoA‐I‐containing HDL. In addition, ApoE deficiency in mice markedly alters the migration pattern of ApoA‐I‐containing HDL in mice, whereas no such effect is observed in humans [6]. ApoE deficiency in mice leads to a marked reduction in phospholipid and cholesterol levels in serum HDL, particularly in large‐sized HDL, and is associated with decreased function, such as macrophage cholesterol efflux capacity [6, 7].

In summary, the finding by Martínez‐Beamonte et al. [1] that dietary supplementation with extra virgin olive oil may enhance HDL function is quite intriguing, but as the authors point out, it will be important in the future, of course, to extend their findings in mice to human subjects.

## Funding

The authors have nothing to report.

## Conflicts of Interest

Masaki Sato, Edward B. Neufeld, and Alan T. Remaley have a patent US20220178953A1; WO2020185598A1. Masaki Sato and Masato Hamasaki are employed by Eiken Chemical Co., Ltd.
